# Laparoscopic augmented reality registration for oncological resection site repair

**DOI:** 10.1007/s11548-021-02336-x

**Published:** 2021-04-02

**Authors:** Fabian Joeres, Tonia Mielke, Christian Hansen

**Affiliations:** Department of Simulation and Graphics, Research Campus STIMULATE, Otto-von-Guericke-University, Magdeburg, Germany

**Keywords:** Augmented reality, Laparoscopic surgery, Partial nephrectomy, Registration

## Abstract

****Purpose**:**

Resection site repair during laparoscopic oncological surgery (e.g. laparoscopic partial nephrectomy) poses some unique challenges and opportunities for augmented reality (AR) navigation support. This work introduces an AR registration workflow that addresses the time pressure that is present during resection site repair.

****Methods**:**

We propose a two-step registration process: the AR content is registered as accurately as possible prior to the tumour resection (the primary registration). This accurate registration is used to apply artificial fiducials to the physical organ and the virtual model. After the resection, these fiducials can be used for rapid re-registration (the secondary registration). We tested this pipeline in a simulated-use study with $N=18$ participants. We compared the registration accuracy and speed for our method and for landmark-based registration as a reference.

****Results**:**

Acquisition of and, thereby, registration with the artificial fiducials were significantly faster than the initial use of anatomical landmarks. Our method also had a trend to be more accurate in cases in which the primary registration was successful. The accuracy loss between the elaborate primary registration and the rapid secondary registration could be quantified with a mean target registration error increase of 2.35 mm.

****Conclusion**:**

This work introduces a registration pipeline for AR navigation support during laparoscopic resection site repair and provides a successful proof-of-concept evaluation thereof. Our results indicate that the concept is better suited than landmark-based registration during this phase, but further work is required to demonstrate clinical suitability and applicability.

## Introduction

Minimally invasive surgical therapy (e.g. laparoscopic surgery) yields multiple clinical benefits over open surgery. However, the surgeons cannot directly access the surgical site in these interventions. This causes several cognitive challenges. Ample research is being conducted to try and mitigate these challenges through image guidance and augmented reality (AR) assistance in laparoscopic surgery [3, 4]. One operation that has attracted wide attention from the research community is laparoscopic or robot-assisted partial nephrectomy (LPN/RPN) [9].

LPN/RPN is an intervention in which localised renal tumours are surgically removed from the kidney while preserving as much healthy kidney tissue and, thereby, renal function as possible [15, 19]. During this operation, three phases can particularly benefit from AR support: (1) the management of renal blood vessels, (2) the intraoperative planning and execution of the tumour resection, and (3) the repair of the resection wound after the tumour has been removed [18]. There are multiple AR solutions proposed in the literature to support surgeons during the first two phases. However, there are no published AR navigation solutions for the third surgical phase [18]. This phase of resection site repair poses some specific challenges for the AR registration and visualisation. AR registration is the correct alignment of the virtual content’s and the physical environment’s coordinate systems. These challenges are discussed below. To our knowledge, no registration concepts exist that aim to support surgeons in the laparoscopic repair of resection wounds.

The resection site repair is conducted under time pressure because it is either conducted under ischemic conditions (if the renal blood vessels have been clamped for resection) or under bleeding (if the vessels have not been clamped). This means that any unnecessary delay increases the risk of renal function loss or blood loss [14, 28]. One potential solution for this might be conducting the registration before the resection and then tracking the kidney during the resection. However, to our knowledge, current organ tracking techniques have not been shown to be robust against resection of major volumes from an organ or loss of sight of the organ surface [20, 30]. Both of these scenarios are realistic during the tumour resection.

Generally, registration methods can be classified as manual, point-based, surface-based, and volume-based methods [3]. Manual (e.g. [22]) and volume-based methods [26] are not suitable for the resection site repair phase because they require too much time. While recent laparoscopic AR registration concepts for LPN/RPN tend to rely on surface point cloud acquisition [10], this method requires general integrity of the organ surface, i.e. it has not been shown to be robust against the resection of an organ volume. Another approach makes use of artificial fiducials on the organ [29], which require intraoperative imaging like computed tomography (CT). In this article, we present a two-step registration approach with artificial fiducials that aims to minimise the registration time during the resection site repair phase without the need for intraoperative imaging.

## Registration method

This introduces a registration pipeline that compromises registration speed and accuracy in the time-critical surgical phase after tumour resection. We propose the use of internal, artificial landmarks that allow for fast point acquisition. The intraoperative placement of artificial markers traditionally requires intraoperative imaging. We propose a two-part registration process to eliminate this need for intraoperative imaging. The first subsection explains the conceptual workflow that we propose. The second subsection describes our prototypical implementation of that workflow. Finally, the experimental methods that we applied for proof-of-concept evaluation are reported in the next section.

### Registration concept

**Fig. 1:**
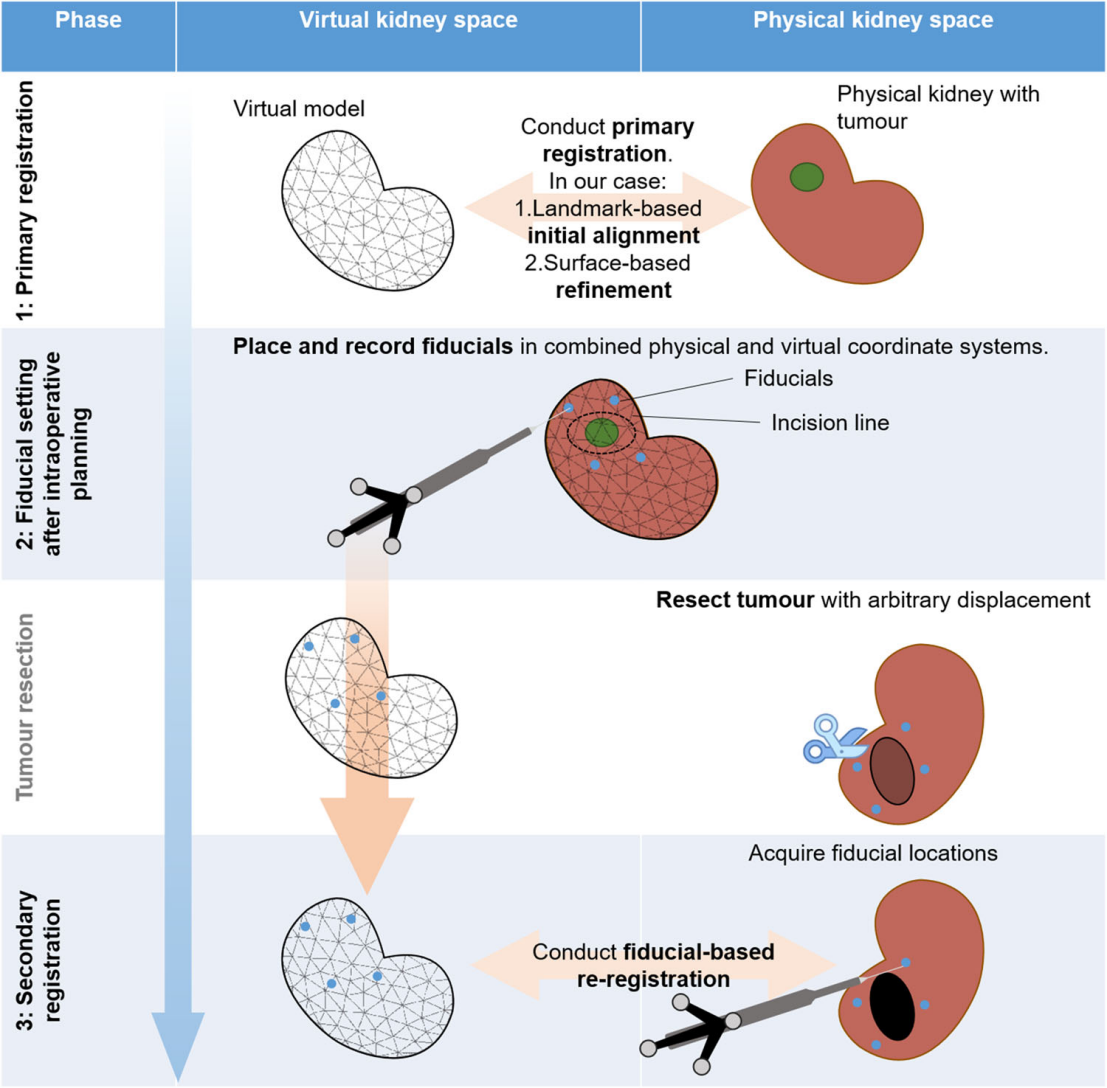
Overview of the proposed two-step registration procedure

The overall two-step registration procedure is summarised in Fig. 1: a primary registration process is completed before the resection is started, but after the intraoperative resection planning is complete. We propose that the primary registration be conducted before vessel clamping to reduce time pressure on this registration procedure. The focus for this registration lies on accuracy rather than speed. This can be conducted by any established means, as described in the literature.

In our case, the primary registration consisted of two steps: an initial alignment and a surface-based refinement step. For initial alignment, we used four anatomical landmarks. For surface-based refinement, we used the Iterative Closest Point (ICP) algorithm [5]. In the clinical application, this may be further refined by non-rigid deformation adaptation. However, this was outside of this work’s scope for reasons discussed further below. Other registration methods may also be used for this primary registration.

After the primary registration is complete, we assume that the virtual and physical kidneys are registered as accurately as possible. The surgeon then places four artificial markers around the planned incision path and, thereby, around the intended resection area. We propose using adhesive markers [29]. These markers’ positions are recorded with an optically tracked pointing tool. The recorded positions are stored in the virtual model for later re-registration. It should be noted that the recorded positions of the tracked pointing tool are situated slightly above the organ surface. This is because the adhesive markers are not thin slices but rather of unknown thickness. We store the position as it is recorded, i.e. slightly above the virtual organs’s surface. Due to any remaining registration errors from the primary registration, the recorded point may be located *below* the virtual model’s surface. In that case, we also store the point as it is recorded. After this step, the surgeons can proceed with the tumour resection, while the artificial markers remain in place.

When the resection is completed, the secondary registration is conducted by the surgeon. At this point, the system’s graphical user interface (GUI) displays the virtual model with the previously recorded points. Following these, the surgeon acquires the artificial markers with the tracked pointing tool. In the concept’s current implementation, the re-acquired points are used for a rigid re-registration. The aim of providing the artificial landmarks during the secondary registration is to increase the speed and accuracy of the landmark identification and, thereby, the point acquisition, compared to “naive” acquisition of anatomical landmarks.

### Prototype implementation

We set up a simulated AR environment to test a prototypical implementation for the proposed registration procedure.

#### AR environment

**Fig. 2:**
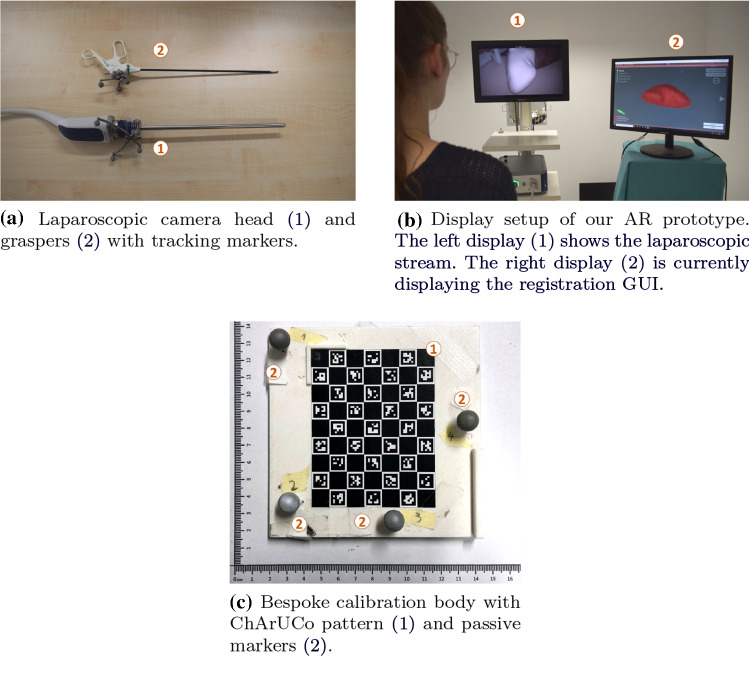
AR prototype components

We implemented a video see-through AR prototype using Unity 2018 (Unity Technologies, USA). The laparoscopic video stream was provided by an Einstein Vision^©^ 3.0 laparoscope (B. Braun Melsungen AG, Germany). This laparoscope was used with a 30°optic in monoscopic mode. The laparoscope’s camera head (Fig. 2a) was optically tracked using an NDI Polaris Spectra infrared tracking camera (Northern Digital Inc., Canada). The laparoscope’s camera was calibrated based on a standard pinhole model’s [31] implementation in the OpenCV^1^ library [6]. We used a ChArUCo pattern [13] for the internal camera parameter calibration and a bespoke calibration body (Fig. 2c) to determine the spatial transformation between the camera head’s marker body and the camera position. Standard laparoscopic graspers (Fig. 2a) served as a generic laparoscopic pointing tool. These were also optically tracked. The transformation between the pointing tool’s marker body and tooltip was determined with a pivot calibration using the NDI Toolbox software (Northern Digital Inc., Canada).

The laparoscopic video stream was duplicated and overlaid with the virtual AR content. A 24$''$ LCD screen displayed either the resulting AR video stream or the registration GUI. The unaltered laparoscopic video stream was permanently displayed on a separate screen (Fig. 2b).

#### Registration interface and workflow

**Fig. 3:**
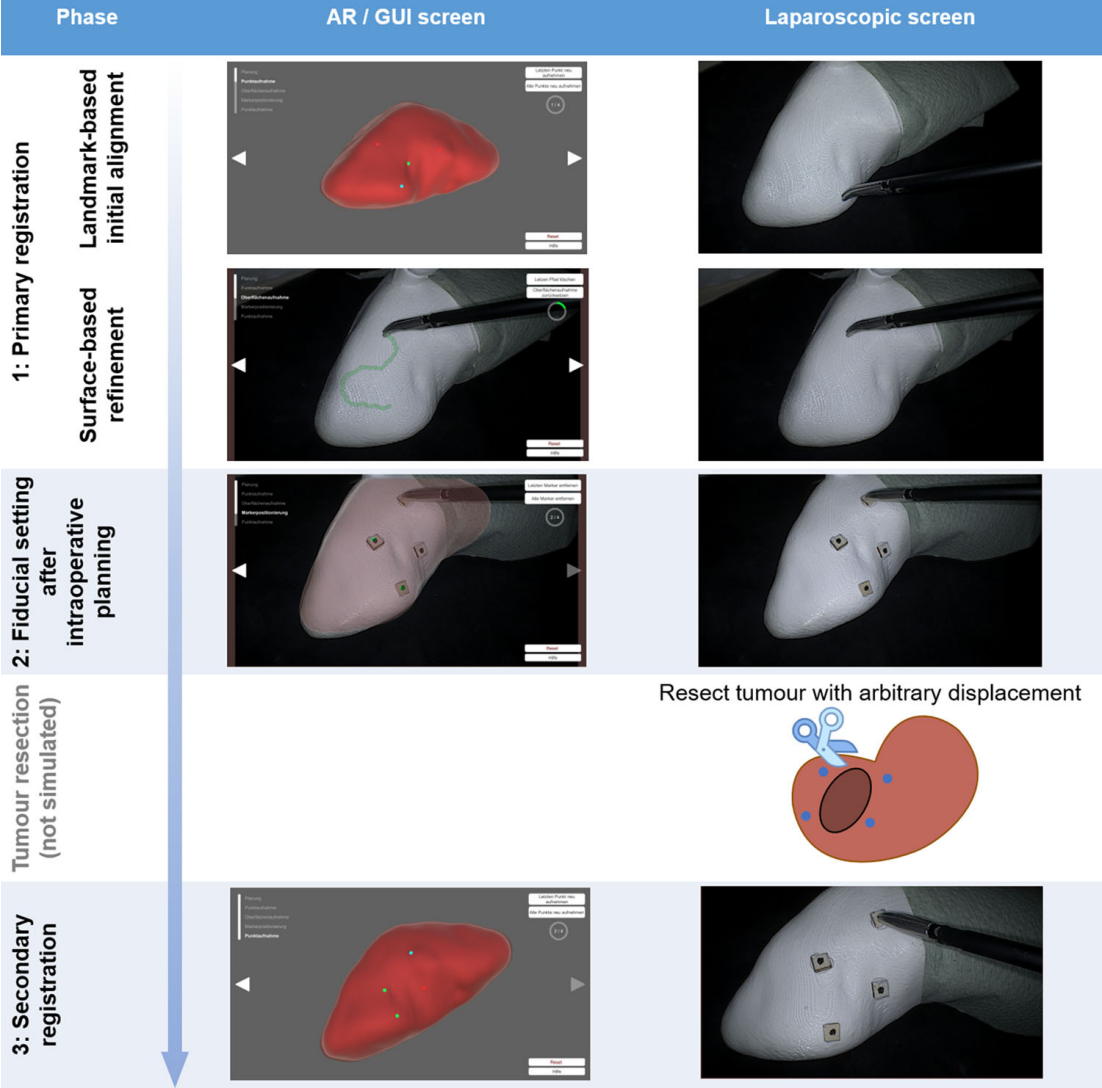
Overview of the prototypical workflow implementation. Participants in our study always saw both screens simultaneously

An overview of the prototypical workflow implementation is provided in Fig. 3. For the initial landmark-based registration, the user was provided with a GUI displaying the virtual model. The user was required to select four characteristic points on the surface with a mouse, as currently applied in clinically used AR systems [8]. Participants were instructed to select characteristic points that they would recognise on the phantom. After this, the points were highlighted one after the other and the user was required to record the points with a spatially tracked pointing tool. The registration transformation was calculated based on the two resulting point clouds [2].

The surface point cloud acquisition was conducted with the same tactile pointing tool: the user was required to trace it across the phantom surface while activating point acquisition with a foot pedal. Points were recorded along this path at 2 mm distance. After at least 200 points had been recorded, the user could trigger the ICP-based registration. There is, to our knowledge, no optimal number of points reported in the literature. However, a range of 40–200 points has been reported for neurosurgery [11]. This step completed the primary registration.

The next step required users to attach simulated adhesive artificial markers to the kidney phantom. These markers aimed to simulate adhesive surgical markers. No specific location instructions were given to the participants. Once completed, the marker positions were recorded and stored in the virtual model. This concluded the simulated workflow that would be expected prior to the resection.

The secondary registration is meant to be conducted after the tumour resection and during the resection site repair phase. At the start of the secondary registration, the user was required to record the marker positions with the tracked pointing tool. The final secondary registration was then conducted based on the two point clouds [2]. This concluded the secondary registration process.

## Experiment methods

We evaluated our two-step registration concept in a simulated-use study. The study aimed to investigate two aspects: firstly, to evaluate whether our method would improve registration speed and accuracy during the time-critical phase as compared to the naive use of anatomical landmarks. Secondly, our study aimed to assess the magnitude of the accuracy loss between the surface-based primary registration and the secondary registration.

### Study design

Regarding the first study objective, we compared registration performance between the initial alignment that was based on anatomical landmarks and the secondary registration that was based on the artificial adhesive fiducials, i.e. we compared the performance at two different stages of the same registration procedure. The independent variable in this aspect was the method applied at the respective stage of the two-step registration process.

We defined four points around each kidney pole that were used as simulated, virtual surgical targets. The first dependent variable was the registration accuracy for these four points, which was operationalised as the mean target registration error (TRE) for these targets. The second dependent variable was the task completion time (TCT) that was required for identifying and recording the landmark points/fiducial positions. Regarding the second study objective, we recorded the difference between the TRE after the completed primary registration and the TRE after the completed secondary registration.

### Sample design

Eighteen participants took part in our study. The participants were medical students in their fourth and fifth year of training. Participants’ age ranged from 21 to 27 years (median $=$ 23.5 years). Twelve participants reported having between 0.5 and 14 h (median $=$ 3 h) of previous experience with laparoscopy (either in clinical applications or in simulators or trainers). We administered some laparoscopic training tasks to mitigate the different levels of prior experience (see Study procedure). Participants were paid 20 EUR for participation.

### Study set-up

**Fig. 4:**
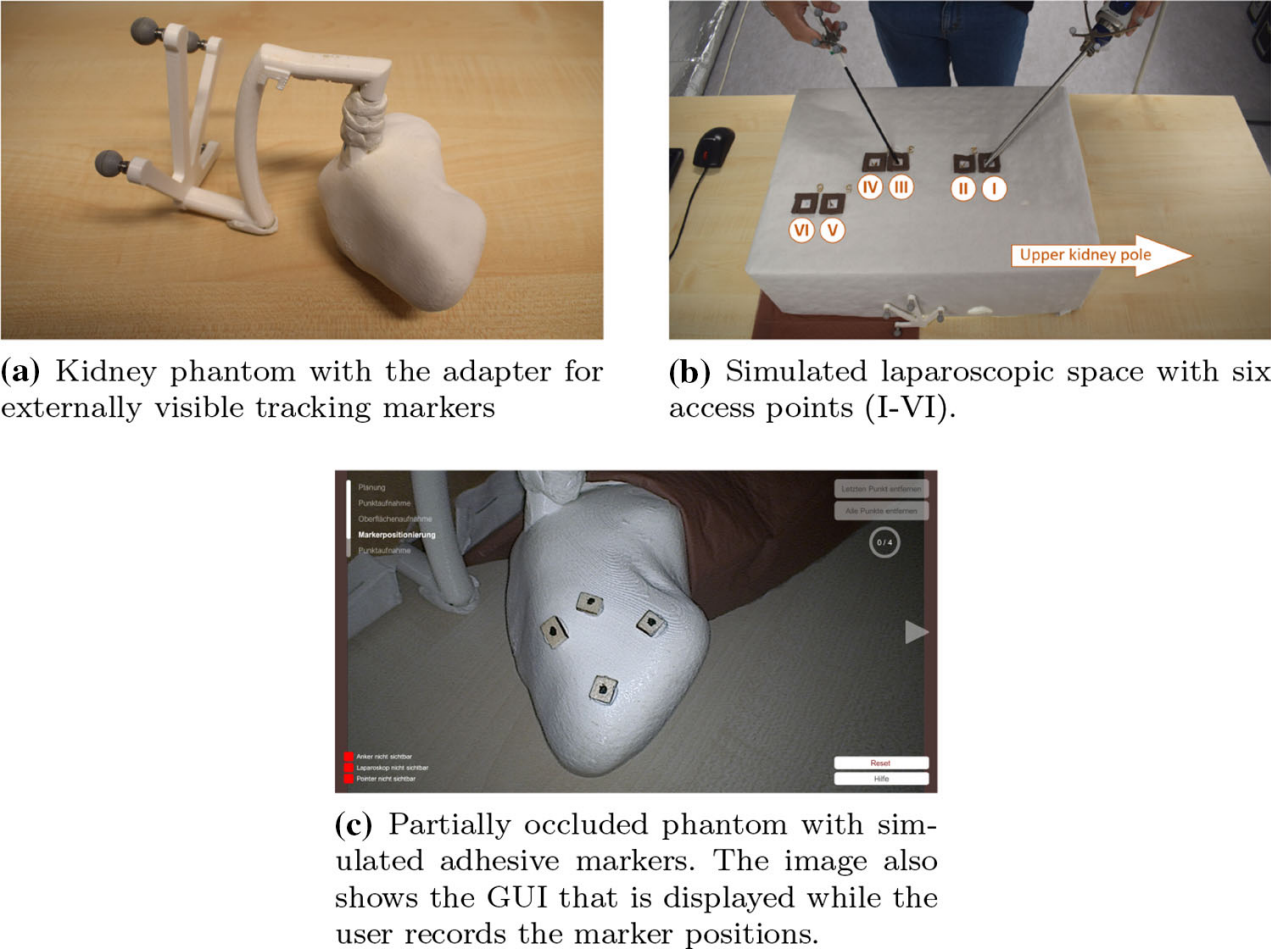
Study set-up components

The surgical site was simulated with a partially occluded phantom. For this phantom, we retrieved a computed tomography imaging dataset of a healthy, adult kidney from a public database [17]. We segmented the parenchymal surface using 3D Slicer [12] and exported a triangle mesh surface model. This model served as the virtual model to be displayed in our AR environment. We prepared a printed phantom of this model with the deposition modelling method. The resulting rigid phantom had a length of 112 mm from pole to pole (original scaling). The phantom was equipped with an adapter in order to spatially track it from outside the simulated surgical site (Fig. 4a).

The phantom was placed inside a simulated laparoscopic workspace. This workspace was created using a cardboard box that occluded the simulated surgical site. The site could be accessed with our tracked laparoscope and pointing tool through six holes in the box (Fig. 4b). The organ motion that would occur in real surgery was simulated by varying the holes through which the workspace was accessed. When the simulated surgical target was on the upper pole (to the participant’s left), holes one and three were used during the primary registration and holes two and four were used for the secondary registration. When the simulated target was on the lower pole (to the participant’s right), holes three and five were used during the primary registration and holes four and six were used for the secondary registration.

**Fig. 5:**
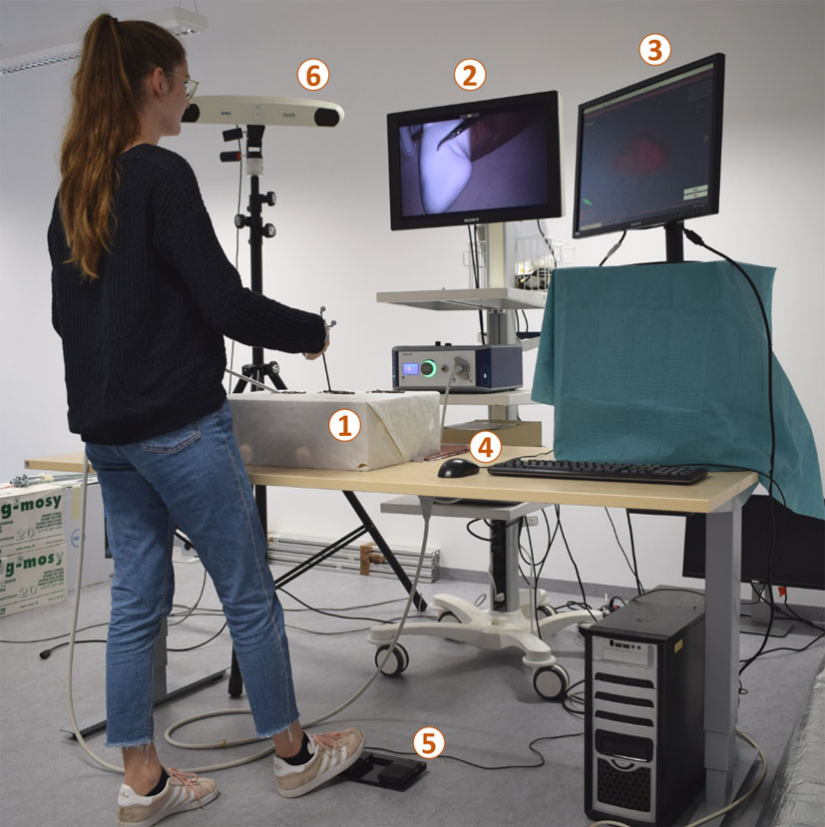
Overall study set-up: (1) simulated laparoscopic environment, including the phantom, camera head, and graspers; (2) laparoscopic screen; (3) AR/GUI screen; (4) mouse for registration planning; (5) foot pedal; (6) optical tracking camera

Approximately half of the phantom was covered with a cloth in each registration procedure. The cloth reached from one of the kidney poles to the phantom adapter (Fig. 4c). The registration was conducted on the non-covered half of the phantom. This aimed to simulate the fact that not the entire renal surface would be revealed during intraoperative dissection. Figure 4c also displays the simulated adhesive markers that were applied in our study. The resulting overall study set-up is shown in Fig. 5.

### Study procedure

We collected participants’ demographic information before the main experiment. Participants were asked to complete two laparoscopic training tasks to practise the particular hand-eye coordination and spatial understanding that are required in laparoscopic interaction. We applied a self-built version of the “bean drop” and “checkerboard drill” tasks [23]. These two tasks require the targeted, coordinated motion of the laparoscope and a laparoscopic tool but are not more complex than necessary for our task (e.g. suturing or cutting tasks). Each task was performed once by every participant. The training performance was not measured or recorded.

**Fig. 6:**
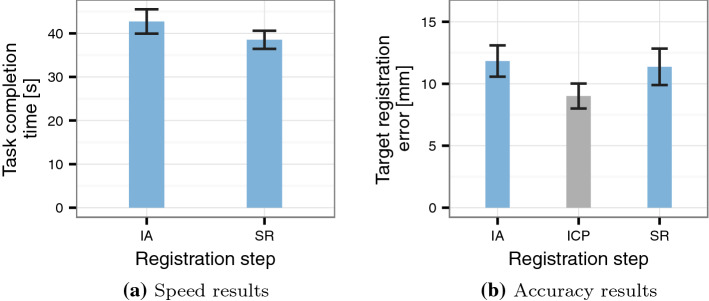
Performance results for the *full sample*. The error bars represent the standard error. IA: initial alignment; ICP: iterative closest point refinement; SR: secondary registration

Following this training, participants conducted the registration process for the first time with step-by-step instructions from the experimenter. They then conducted a second training trial without explicit instructions but with the opportunity to ask questions. After all questions had been answered, the experimenter exchanged the targeted kidney pole (by moving the cloth). Finally, participants performed the registration process in a test trial in which we recorded the required data. This concluded the experiment.

### Hypotheses and data analysis

We conducted one-sided paired *t* tests for the TRE and TCT. The tests compared data for the initial landmark-based registration (prior to surface-based refinement) and the secondary registration with the alternative hypotheses:

1 $$\begin{aligned}&H_{1,\mathrm{TRE}}: \mathrm{TRE}_\mathrm{secondary\,\, registration} < \mathrm{TRE}_\mathrm{initial \,\,alignment} \end{aligned}$$

2 $$\begin{aligned}&H_{1,\mathrm{TCT}}: \mathrm{TCT}_\mathrm{secondary\,\, registration} < \mathrm{TCT}_\mathrm{initial\,\, alignment} \end{aligned}$$

It is inherent in our concept that the TRE will systematically increase between the refined primary registration and the secondary registration because the latter builds on the former. We, therefore, did not perform significance tests for this difference but rather identified confidence intervals to provide an estimate for the magnitude of the accuracy loss during this step. Modified post hoc tests were conducted as reported in the Results section.

## Results

The point acquisition phase could be conducted significantly faster during the secondary registration than during the initial alignment ($T=1.80, p=0.045$, Fig. 6a). The registration accuracy was not significantly higher across the full sample ($T=.025, p = 0.402$, Fig. 6b).

The mean TRE difference between the primary surface-based registration and the secondary registration amounted to 2.35 mm (CI_95_ $=$ [0.47 mm, 4.23 mm]).

### Data exclusion and post hoc analysis

**Fig. 7:**
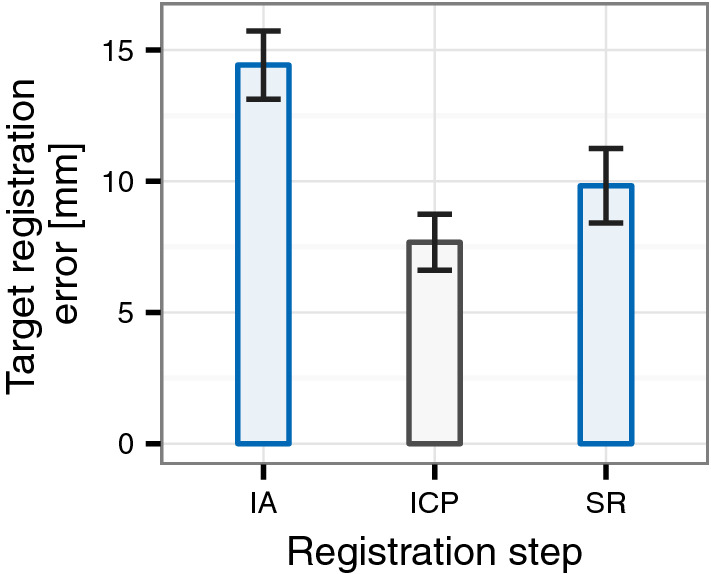
Accuracy results for the *reduced sample*. IA: initial alignment; ICP: iterative closest point refinement; SR: secondary registration

Generally, the surface-based registration step is conducted to refine the landmark-based initial alignment. It is generally expected to increase registration accuracy [24, 27]. However, data analysis showed that six of our participants produced a higher TRE during surface-based refinement than during the initial landmark-based registration. We believe that this registration error increase during what is intended as a registration refinement step is likely to be caused by a number of errors that are described in Discussion section below. These errors are unlikely to be encountered by experienced surgeons, i.e. the intended user population for systems like this. We, therefore, excluded these six participants for a post hoc exploratory and descriptive analysis of the TRE development. The resulting reduction is shown descriptively in Fig. 7.

## Discussion

### Discussion of results

Our results indicate that the two-step registration solution can improve registration speed and may be able to improve accuracy for laparoscopic AR applications in time-critical surgical phases. One limitation of our results is the unusually low overall accuracy of the surface-based primary registration. We see two potential reasons for this: firstly, we used pivot-calibrated standard laparoscopic graspers as an optically tracked tactile pointer to record the required point clouds. We chose this instrument because it is readily available in the operating room. However, it is somewhat flexible and bends easily under mechanical load. This considerably affects the tooltip tracking as that is based on a rigid pivot calibration. An interesting follow-up objective of our work may lie in the measurement and quantification of this deformation and its contribution to the overall registration error. To our knowledge, this has not been previously reported in the literature. Secondly, our participants had very limited experience with handling laparoscopic tools. We anecdotally observed that several participants accidentally recorded some points after the tooltip had slipped off the phantom surface. Participants also applied high pressure when tracing the instrument across the phantom surface, which increased the issue of instrument deformation. Moreover, it was difficult for some participants to keep the tooltip rather than the side of the tool on the surface because of the typically constrained tooltip motion. It seems unlikely that experienced laparoscopic surgeons would experience these specific difficulties.

Inaccuracies in the surface-based primary registration are passed on to the secondary registration. This is because the fiducial location in the virtual model is conducted after and, thereby, based on the primary registration. Thus, there is a discrepancy between the physical fiducial’s position on the kidney and the fiducial’s recorded position on the virtual model. This “fiducial storage error” is added to the fiducial localisation error that occurs during the secondary point acquisition. We were able to quantify the impact of the resulting accuracy loss (fiducial storage error plus fiducial localisation error) with a TRE growth of approximately 0.47–4.23 mm. We, therefore, believe that absolute accuracy could be considerably improved by modifying the means of surface acquisition.

### General discussion

While this article represents a successful proof-of-concept evaluation for our two-step registration method, it does not yet demonstrate clinical applicability or benefit. The obvious follow-up question for our results is whether the improvements that our method brings are sufficient to make AR support feasible during the time-critical phase of resection site repair. Specifically, three questions arise: firstly, is the added registration task with an estimated duration of 40 s during a time-critical phase justified by the clinical benefit? That is, can the resection site repair either be accelerated enough to compensate for the additional 40 s or does the additional information make the process more safe and effective? Future work should also examine whether this can be further accelerated by supporting the user in the fiducial acquisition. For example, the fiducials could be detected and highlighted in the video stream. Secondly, is the registration (with improved surface acquisition accuracy) sufficiently accurate to provide meaningful information about the position of risk structures? Finally, our participants’ experience does not reflect the skill level of the experienced surgeons that would use the system in a real application. The different levels of experience may influence users’ abilities to recognise landmarks/fiducials due to a better understanding of the surgical site and to record those landmarks/fiducials due to a higher skill level by using the laparoscopic tools. The third question is, therefore: Which accuracy levels would experienced surgeons achieve with this approach? These questions remain to be answered in future follow-up work.

Another important scope limitation is that we did not consider the effects of organ deformation during the tumour resection. Organ deformation in abdominal AR registration is a major limiting challenge and an active field of research [1, 21]. Promising concepts exist in the literature to mitigate this by applying biomechanical models to the virtual content and, thereby, simulating the physical organ’s deformation. One approach [20] informs a biomechanical model via fiducial marker locations and is, therefore, promising for our application and marker-based concept. However, to our knowledge, current biomechanical models [20, 30] assume that the kidney is deformed but structurally intact. In our application, however, the kidney is additionally deformed from its preoperative state by removing an unknown tissue volume. While some data have been published on the surface deformation caused by a single straight-line incision [1], a biomechanical model for our application would also have to consider the intrarenal structure deformation that is caused by the removal of a tissue sample. While this requires further research, a deformation study for the liver [16] has shown that intraoperative deformation is very limited on a local scale. Thus, within the area of the four fiducials and resection site, rigid registration may even prove to be sufficient.

The two conditions that we compared in our study were measured in a fixed order. This may have led to training effects between the two stages of the registration process. Specifically, participants were more familiar with the surgical object (in our case, the phantom) during the secondary registration than during the primary registration. A part of the fiducial acquisition acceleration may be attributed to this fact. However, this prior familiarisation with the surgical site is realistic and, therefore, does not affect the validity of our results.

Overall, registration accuracy in a clinical setting may be higher due to better surface acquisition methods, or it may be lower due to organ deformation. Thus, the absolute TRE values from our study are of limited external validity. However, the effects we found indicate that our concept may be a viable approach for AR support during the resection site repair phase of LPN/RPN.

It should be noted that this article presents a registration concept for the resection bed but does not address the issue of visualising relevant anatomical information. This poses a separate challenge because the exact resected volume is unknown at this point during the surgery. Further work is required to address this, but one potential approach may be visualisations that are based on instrument locations (e.g. [25]) rather than the permanent bulk display of anatomical structures (e.g. [7]).

This article discusses the proposed registration method in the context of resection wound repair in LPN/RPN. Further research is required to assess its suitability in other laparoscopic oncological resections. Moreover, the general two-step concept may be suitable for even more image-guided surgery applications in which the registration process is conducted under time pressure and in which the opportunities for intraoperative imaging or preoperative fiducial placing are limited.

## Conclusion

This work introduces and evaluates a two-step registration method with artificial adhesive fiducials for AR in laparoscopic resection wound repair, with the example of LPN/RPN. Specifically, the method aims to reduce the required registration time for AR support during this surgical phase. Our results show that the method is faster and has the potential to be more accurate than other landmark-based methods and that it is faster than surface-based registration. While the results do not finally demonstrate clinical applicability, they represent a proof of concept for our two-step registration method. Further research is required to investigate the tissue deformation during tumour resection in order to achieve clinical feasibility for any post-resection registration approach. Moreover, dedicated visualisation methods for this AR application are yet to be developed. Overall, we believe that the work presented in this article is an important stepping stone towards providing AR navigation support during the resection site repair in LPN/RPN and potentially even other laparoscopic surgical interventions.

## Research ethics

Written informed consent was obtained from all individual participants before study commencement. All procedures performed in our study were in accordance with the 1964 Helsinki declaration and its later amendments. Institutional review and approval were not required due to the non-invasive nature of our study.

## Footnotes

1 We used the commercially available *OpenCV for Unity* package (Enox Software, Japan).
